# Professional Olla-Podrida

**Published:** 1872-03

**Authors:** Robert Battey

**Affiliations:** Rome, Georgia


### PROFESSIONAL OLLA-PODRIDA.

BY ROBERT BATTEY, M.D., ROME, GEORGIA.

*Editors Atlanta Medical and Surgical Journal:*

You will, perhaps, recall the fact that I contributed to your journal, some years ago, a series of papers headed as above. I know not how I can better fulfill my promise to aid you in your commendable enterprise than by continuing the series of thoughts, observations, experiences, suggestions, speculations, etc., which made up my olla-podrida.

Anomalous Pulse.—On the 3d October last, my son Henry, a boy of fourteen, was seized of typhoid fever, and passed through an ordinary, rather mild attack. On the 20th, he seemed quite convalescent, with good appetite, and had been clear of fever for several days. But on my return home at night, I was surprised to find him with a pulse of forty-four beats per minute—the skin bathed in cool, clammy sweat—the tongue red, slick, and rather dry. He was stimulated, the skin well-rubbed off, and passed a quiet and comfortable night. On the 21st, the pulse stood quite constantly at fifty-two; tongue less red and more moist; appetite fair. On the 22d, the pulse ranged from forty-six to fifty-two; on the 23d, from fifty-two to fifty-six; 24th, the same; the tongue is now quite natural, appetite good. 25th, pulse fifty-six to sixty; bowels rather too loose. 26th, pulse forty-eight to fifty-two. 27th, pulse sixty; seems brighter; bowels still too loose. 28th, pulse, morning and evening, at forty-four; does not seem so well. 29th, pulse forty-eight in morning, forty-two in afternoon; good appetite. 30th, pulse forty-two to forty-four; seems stronger; bowels still loose. 31st, pulse forty-four to fifty-two; sat up a little to-day. November 1st, pulse sixty in the morning, fifty-two at noon, and forty-eight in the evening; an exanthematous rash has appeared upon the surfaces generally. 2d, pulse sixty-four; is improving; sits up some. 3d, pulse seventy-eight; there is œdema of face and feet. 4th, pulse sixty; œdema increased; urine scanty, and highly colored. 5th, the same; sits up several hours. 6th, urine slightly sanguineous, containing albumen. 7th, pulse seventy; œdema continues; appetite good. 12th, œdema is much increased; pleuritic effusion; respiration embarrassed; has some fever; at night the breathing was much labored; distressing cough; rusty, pneumonic expectoration. 13th, better; the cough is gone; breathes much easier; appetite is good. 14th, still better; breathes easy; limbs still much swollen. 15th, gaining ground; breathes normally; urine sanguineous, and loaded with albumen. 21st, urine abundant and less sanguineous; free perspirations at night; respiration easy; œdema of the legs going down. 25th, urine abundant and clear; œdema is nearly all gone; sits up all day, and moves about the house.

*Remarks.*—I have never before observed so great and long-continued depression of the pulse. The nearest approximation to it which has come under my eye was the case of George R——, a youth of thirteen, with bilious-remittent, in which, for three days, the pulse went down to sixty during the daily paroxysm of fever, and rose to seventy-six as the fever subsided. In no other respect did it differ from ordinary remittent, and was of but few days' duration. In two subsequent attacks, with George, the pulse anomaly did not appear.

Chloral.—Amongst all the more recent contributions to our *Materia Medica*, since the introduction of chloroform, there is, perhaps, none comparable in value to chloral. It is unfortunate that the quality of it, as dispensed in the shops, is so variable in physical properties and medicinal effects. I have used the German preparations in the form of flat cakes, of irregular masses, and of crystals—also the manufacture of Powers & Wightman—but have found none so uniform and satisfactory in every way as that made by Dr. Squibb, of Brooklyn, New York.

I am in the habit of dispensing it to my patients in the form of a standard solution of one drachm to the ounce of water—of which a teaspoonful, representing seven and a half grains of chloral, is the usual dose. Ordinarily I have not had occasion to increase this dose. In one instance only have I allowed a patient to take it in doses of thirty grains; but in no case do I permit more than sixty grains in the day. Right sorry am I that certain gentlemen at a distance have been led to give doses of one drachm and upwards, with now and then fatal results, and thus got the matter into the newspapers, and rendered my patients nervous at the bare mention of chloral. It deprives me, sometimes, of the opportunity of doing great good with it, as I believe.

I have not found chloral, alone, very effective in the relief of pain; but if a dose of one or two teaspoonsful of my solution be given, and the patient inhale afterward a little chloroform vapor, to lull the pain until sleep comes on, the return of pain does not usually arouse the slumberer.

The pangs of parturition are greatly mitigated by small doses of chloral, and the nervous system braced up advantageously for the conflict. In natural labor, when anything is needed, it seems to me decidedly more advantageous than chloroform. I have not found it so apt to induce uterine inertia and post-partem hemorrhages.

The first impulse with many patients, on swallowing a dose of chloral, is to vomit; but if an effort of restraint is made, and the medicine retained for a few moments, it soothes and composes an irritable stomach.

An Empty Stomach Can't Vomit.—A commonplace truism you call it. Yes, so it is; but how many people, think you, really *know* it? How many learned physicians ever, think you, know it practically at the bedside of the patient? How often have we seen the brains of really good practitioners racked and tortured for new expedients—the leaves of the *Materia Medica* and various works upon therapeutics industriously thumbed for new remedies—when ten, fifteen, yes, twenty, had already failed to allay the upheavings of a rebellious stomach! In years gone by, I too went through this racking and thumbing process, and at last sought the advice of a senior medical friend, now an eminent practitioner of your city. "Why, Doctor, an empty stomach *can't* vomit," was his laconic reply. It rings in my ears as clearly and distinctly now as it did then, and will continue to do so as long as I have strength to feel a pulse.

What a flood of light those few simple words let in upon my mind for the management of my case. It was an octavo volume uttered in a single breath. Do you call it commonplace? and does everybody know it? I failed to learn it on the lecture benches, in the college clinics, at the hospitals. I have searched for it industriously through long years in my well-thumbed library and in the medical journals. I have listened for it from the lips of men high in the ranks at home and abroad, and never, never but once, have I heard the great truth uttered by mortal man.

It is "a small affair," say you? Pardon me, I don't think so. A fundamental truth is always a big thing; and a truth the possession of which transforms me from a nervous, doubting and disappointed mixer of medicaments and simples at the bedside of a vomiting, retching patient, into a quiet and composed *master of the situation*, can never be a small affair with me. It would have done you good to have seen my countenance brighten with hope and conscious power when this light first broke in on me; and it would do you good now if you could see, as I do from year to year, the joy and gladness with which others of the brethren receive it at second hand in their hour of weakness and of need.

How long can a patient live with a stomach absolutely empty? Sixty hours at least, as I can certify, without either food or drink, if only properly nourished by the rectum. Long enough to quiet gastric excitement and secure tolerance for food and medicines.

---

Preparation of Hydro-Sulphuric Acid.—A new and convenient process for procuring hydrô-sulphuric acid is described by J. Galletly, in the Chemical News. It consists of simply heating in a flask a mixture, in equal parts, of paraffin and sulphur. The delivery tube should contain a little loose cotton wool to intercept any sublimed sulphur. The stream of gas may be stopped and renewed at pleasure by withdrawing or applying the heat. Heavy paraffin oil, such as is used for lubricating purposes, or commercial stearic acid may be substituted for the paraffin, but in the latter case a milky fluid, consisting probably of water and finely divided sulphur, condenses in the delivery tube.

